# Progress and Perspectives on Heat Transfer Design Optimization of Functionally Graded Materials Under Large Temperature Gradients

**DOI:** 10.3390/ma19040788

**Published:** 2026-02-18

**Authors:** Fang Zhang, Yifu Shen, Haiou Yang

**Affiliations:** 1 College of Materials Science and Technology, Nanjing University of Aeronautics and Astronautics, Nanjing 211106, China; 2 AVIC Metal Test Technology Co., Ltd., Xi’an 713700, China; 3 Shenzhen Research Institute of Northwestern Polytechnical University, Shenzhen 518057, China

**Keywords:** functionally graded materials (FGMs), large temperature gradient, heat transfer optimization, structural optimization, topology optimization, numerical simulation

## Abstract

Large temperature gradients encountered in aerospace, energy, and microelectronics systems impose stringent requirements on material thermal performance. Functionally graded materials (FGMs), characterized by a continuous variation in composition and properties, offer significant advantages in regulating heat transfer and mitigating thermal stresses. This review provides a systematic summary of recent progress in heat transfer design optimization of FGMs under large temperature gradient conditions. From a methodological perspective, advancements in structural and compositional optimization, topology optimization, and multi-objective optimization are reviewed. Numerical simulation techniques, including conventional finite element and finite volume methods, as well as emerging approaches such as peridynamics, isogeometric analysis, and meshfree methods, are discussed with an emphasis on multiphysics coupling. In addition, representative applications of FGMs in electronic thermal management, aerospace thermal protection, energy systems, and building energy conservation are reviewed. Current challenges, including idealized modeling assumptions, limited coordination among multiple optimization objectives, and insufficient reliability evaluation in complex service environments, are identified. Finally, future research directions are outlined, highlighting intelligent design methods, multiscale modeling, advanced manufacturing technologies, and multifunctional integration. This review seeks to provide a comprehensive reference for both fundamental research and engineering applications of heat transfer optimization in functionally graded materials.

## 1. Introduction

In modern engineering applications, particularly in high-technology fields such as aerospace, energy systems, and microelectronics, materials are exposed to increasingly complex thermal environments. Extreme service conditions involving large temperature gradients present severe challenges to material performance. Large temperature gradients are defined as temperature differences within a material or across a system that are substantial enough to induce significant variations in thermal stresses, nonlinear heat transfer behavior, and temperature-dependent material properties [1]. These gradients are commonly encountered in high-power electronic devices, aerospace components, and energy systems, where temperature differences of several hundred degrees Celsius can exist over short distances [2]. Under such conditions, the thermal response becomes highly nonlinear, and material properties, such as thermal conductivity and expansion, may change significantly with temperature. The term “large temperature gradient” refers to thermal gradients generally exceeding 10^3^–10^6^ K/m, which are frequently encountered in microscale electronic cooling, aerospace thermal protection systems, and high-temperature energy devices [3]. Such gradients are highly dependent on application and length scale, and their magnitude can vary significantly across different engineering scenarios. Under these conditions, temperature-dependent material properties, nonlinear heat conduction, and strong thermo-mechanical coupling effects become non-negligible, fundamentally distinguishing large-gradient heat transfer problems from conventional moderate-gradient cases [4]. It should be noted that the duration over which large temperature gradients can be sustained without inducing structural deformation or damage is not solely governed by heat transfer. Instead, it is controlled by coupled thermo-mechanical behavior, including thermal stress evolution, creep, fatigue, and potential material degradation under prolonged thermal loading [5,6]. Consequently, the allowable exposure time to large temperature gradients is highly problem-specific and is typically evaluated through combined thermo-mechanical simulations and experimental assessments rather than by a universal criterion.

Due to their unique characteristics, functionally graded materials (FGMs) have been recognized as an ideal solution to address these challenges [7]. FGMs are composite materials in which at least one material property—such as composition, microstructure, elastic modulus, or thermal conductivity—varies continuously according to predefined functions (e.g., linear, exponential, or periodic distributions) within the material domain. These materials are widely regarded as effective in alleviating thermal stresses and tailoring thermal barrier properties [8]. Compared with conventional homogeneous materials, FGMs enable spatially continuous property transitions, effectively mitigating stress concentrations and enhancing durability and reliability. In particular, under large temperature gradient conditions, the graded structural design of FGMs significantly reduces thermal stresses, improves thermal shock resistance, and extends service life [9]. From a physical perspective, the effective heat conduction pathways within the material may be significantly altered by spatially varying thermal conductivity, while thermal resistance redistribution occurs due to gradient-induced changes in material composition and microstructure. These phenomena are particularly pronounced in functionally graded materials, where the intentional spatial variation in properties provides additional degrees of freedom for regulating heat flow and mitigating thermal stresses.

Nevertheless, fully exploiting the heat transfer potential of FGMs under large temperature gradients requires thorough investigation and systematic optimization of their thermal design [10]. Heat transfer design encompasses multiple aspects, including material composition distribution, microstructural configuration, and gradient profiles, and must simultaneously account for various heat transfer mechanisms such as conduction, radiation, and convection [11]. Moreover, the evolution of physical and chemical properties under severe thermal gradients, along with the resulting stress distribution and damage mechanisms within FGMs, adds complexity to heat transfer optimization. The effective heat conduction pathways in functionally graded materials (FGMs) play a crucial role in optimizing thermal performance under large temperature gradients. By tailoring the material composition and microstructure, FGMs can direct heat flux along preferential channels, effectively reducing thermal resistance and enhancing heat transfer. These materials facilitate a smooth transition of thermal conductivity across different regions, which is essential for alleviating thermal stresses and preventing thermal shock under high thermal gradients. The design of FGMs aims to mitigate localized temperature spikes by redistributing thermal resistance, thereby ensuring more uniform heat flow across the structure. This characteristic of FGMs is particularly beneficial in applications such as electronic cooling, where heat dissipation must be optimized, and aerospace systems, where the material must endure extreme thermal loads.

In recent years, significant progress has been made in various heat transfer optimization strategies for FGMs, including structural and compositional optimization, topology optimization, and multi-objective optimization approaches. Concurrently, various numerical simulation techniques—such as the finite element method, boundary element method, and meshfree methods—have been widely used to analyze heat transfer behavior in FGMs. In addition, optimized thermal designs of FGMs have been actively explored across various application scenarios, including electronic cooling systems and aerospace thermal protection structures.

Accordingly, this review systematically summarizes recent advances in heat transfer design optimization of FGMs under large temperature gradients. Emphasis is placed on optimization methodologies, numerical simulation techniques, and representative application scenarios. This review aims to summarize recent advances in heat transfer design and optimization of functionally graded materials under large temperature gradients, focusing particularly on optimization methodologies and numerical frameworks. Beyond a descriptive survey of existing studies, the review aims to extract common physical trends and governing mechanisms underlying heat transfer regulation in FGMs, especially in relation to gradient design, effective heat conduction pathways, and thermal resistance redistribution. Unlike existing review articles that primarily classify FGMs according to fabrication techniques or material systems, the present review is organized from the perspective of heat transfer optimization under large temperature gradients. The literature is structured according to optimization strategies and representative engineering application scenarios, with an emphasis on how gradient design, topology optimization, and multi-objective formulations influence thermal performance. This organization aims to provide an integrated understanding of the design logic behind heat transfer optimization of FGMs and to facilitate cross-comparison among different application-driven studies. In this review, ‘heat transfer optimization’ primarily refers to the design of functionally graded materials (FGMs) to optimize heat conduction efficiency and minimize thermal resistance. While studies involving thermally induced mechanical responses (e.g., thermal stress, deformation) are discussed, these are considered secondary effects influenced by the primary thermal field and are addressed only when they directly impact the thermal performance of FGMs. Finally, future research prospects are discussed, aiming to provide guidance and references for both fundamental studies and practical engineering applications of heat transfer optimization in functionally graded materials.

## 2. Heat Transfer Design Optimization Methods for Functionally Graded Materials

### 2.1. Structural Optimization Design

The structural optimization design of functionally graded materials (FGMs) plays a critical role in enhancing their thermal performance. Consequently, extensive research efforts have focused on integrated optimization strategies for FGMs to achieve high-performance heat transfer characteristics [12]. Through the rational design of structural parameters, optimal material configurations can be obtained. This process primarily involves the optimization of key parameters, such as gradient function forms, layer distributions, and thickness ratios, and is often addressed using multi-objective optimization approaches, particularly when temperature-dependent material properties must be considered [13]. These parameters directly influence thermal conductivity, thermal stress distribution, and the overall thermo-mechanical performance of FGMs. The material composition distribution and gradient profiles directly influence thermal conductivity and heat flow pathways in FGMs, which in turn affect the thermal stress distribution. While the thermal stress response is often a result of the temperature gradient, it is primarily addressed as a secondary factor in optimizing heat transfer performance.

The optimization of gradient function forms constitutes a core aspect of FGM structural design. FGMs typically consist of two or more constituent materials, whose chemical composition, microstructure, and porosity vary continuously along a prescribed direction, resulting in specific gradient distribution functions [14]. Commonly adopted gradient functions include power-law distributions [15], exponential distributions [16], and linear distributions [17]. In recent years, numerous studies have demonstrated that different gradient functions can significantly affect temperature field distributions, heat flux density, and overall heat transfer efficiency.

**Figure 1:**
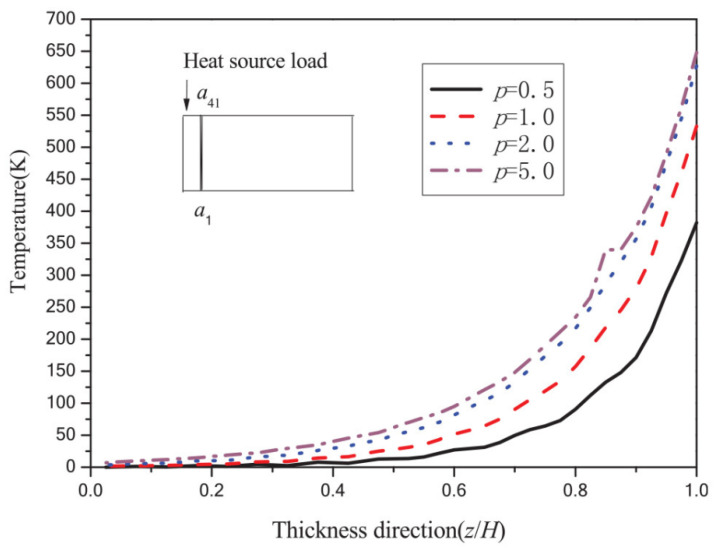
Temperature distribution of the thickness direction with the different gradient parameters. Reprinted with permission from Ref. [18]. Open access.

Tian et al. [18] performed steady-state heat conduction analyses of FGM plates with variable gradient distributions. The temperature distributions along the thickness direction under different gradient parameters are shown in Figure 1. The abscissa represents the normalized thickness coordinate (z/H), where z/H = 1.0 corresponds to the upper surface of the FGM (pure ZrO_2_, 100% ceramic) and z/H = 0.0 corresponds to the lower surface (pure Ti6Al4V, 100% metal). The curves labeled with different values of *p* (*p* = 0.5, 1.0, 2.0, and 5.0) represent temperature distributions corresponding to different gradient parameters. The symbols “a” and “a_41_” in Figure 1 denote observation points defined at x = 0.2H, i.e., at a distance equal to 0.2 times the total thickness H from the heat source along the x-direction. Specifically, point a_41_ is located at the upper surface (z/H = 1.0, pure ZrO_2_), whereas point a is located at the lower surface (z/H = 0.0, pure Ti6Al4V) within the a_i_ series of observation points. Figure 1 clearly demonstrates that the form of the gradient function directly governs the temperature distribution and thermal gradient across the thickness, explaining why gradient-function optimization constitutes a primary design variable in heat transfer optimization of FGMs under large temperature gradients. The results further indicate that variations in gradient function parameters markedly influence both the temperature distribution and temperature gradient across the thickness, confirming that material property gradient forms are key factors governing heat conduction behavior.

Yevtushenko et al. [19], based on a frictional heating model, analyzed the thermal response of FGMs with exponentially varying thermal conductivity and found that exponential gradients can effectively regulate the maximum surface temperature and heat flux distribution, exhibiting favorable temperature control and thermal resistance characteristics. Subsequently, Yevtushenko et al. [20] further investigated coating–substrate systems with different gradient distributions and reported that exponentially graded thermal conductivity significantly reduces peak surface temperatures, thereby enhancing the protective capability of thermal barrier structures. Sobamowo et al. [21] proposed a nonlinear transient heat transfer model for FGM fins with linear and exponential gradient functions and demonstrated that both the gradient form and gradient parameters exert pronounced effects on temperature gradients and heat transfer efficiency, with exponential distributions showing superior thermal performance. Han et al. [22], by employing a thermomechanically coupled finite element–discrete element model, systematically compared the heat conduction behavior and thermal crack evolution of FGMs with linear, quadratic, and exponential gradients. Their findings indicated that variations in gradient function forms directly affect thermal stress distributions and crack propagation paths.

Layer-number optimization represents another important aspect of FGM structural design. Although ideal FGMs exhibit continuously varying material properties, practical engineering applications often approximate continuous gradients using multilayered discrete structures [23]. The number of layers significantly influences temperature distribution, thermal stress levels, and fabrication complexity. Studies have shown that while a smaller number of layers can simplify manufacturing processes and reduce processing time and cost, the resulting property transitions are often not sufficiently smooth, leading to pronounced thermal stress concentrations at layer interfaces and potentially compromising service life [24].

**Figure 2:**
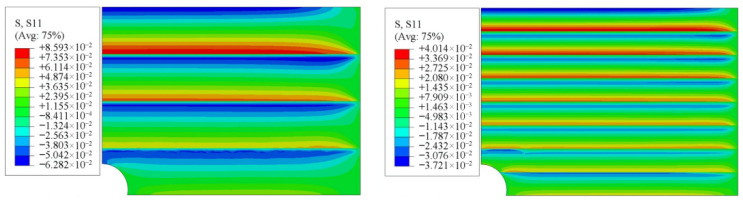
Stress components $\sigma_{\mathrm{xx}}$ (S11) on the distribution of finite element models with 4 and 8 layers. Reprinted with permission from Ref. [27]. Copyright ©2024, Springer Nature.

Dose et al. [25] approximated a continuous gradient using a multilayer structure when designing FGM interlayers to reduce thermal stresses in high-temperature components. Their results indicated that structures with fewer layers exhibit discontinuities in temperature and stress fields, whereas increasing the number of layers leads to smoother thermal stress distributions, bringing them closer to the ideal continuous gradient state. Similarly, Szlachetka et al. [26] investigated heat conduction in stepwise multi-component FGMs and reported that as the number of layers increases, heat flux density and temperature gradient distributions gradually approach those of continuous models. However, excessively increasing the number of layers substantially increases manufacturing complexity and computational cost. Baytak et al. [27] conducted thermal stress analyses of layered FGM plates and found that increasing the number of layers reduces peak interfacial thermal stresses. In Figure 2, the applied heat flux is imposed along the thickness (*z*-axis) direction, resulting in the development of the normal stress component σ_xx_ (denoted as S11 in the finite element output), while simultaneously imposing higher requirements on fabrication precision and interlayer bonding quality. Figure 2 demonstrates the trade-off between thermal stress mitigation and structural discretization, emphasizing that layer-number optimization is essential for balancing thermal performance and manufacturability in practical FGM designs. Therefore, a balance between thermal performance and manufacturing feasibility must be achieved, often through optimization algorithms to determine the optimal layer configuration.

Thickness optimization is equally crucial for the heat transfer performance of FGMs. Due to differences in thermal conductivity and thermal expansion coefficients among constituent layers, variations in layer thickness ratios directly alter heat transfer pathways and effective thermal resistance distributions, thereby influencing internal temperature gradients and thermal stress levels [28]. Many studies have demonstrated that rational design and optimization of layer thicknesses can effectively control heat flow and minimize thermal stresses.

Jabbari et al. [29] investigated the thermomechanical behavior of rotating functionally graded disks and found that thickness distributions play a significant role in determining temperature gradients and thermal stress distributions. Thicker regions provide more effective heat dissipation pathways, while abrupt thickness variations may induce stress concentrations and local buckling risks. Chen et al. [30] employed a genetic algorithm to optimize material composition and geometric parameters of FGM coating systems under thermal loading, demonstrating that appropriate adjustment of layer thicknesses and composition ratios can substantially reduce peak interfacial thermal stresses and improve heat flow uniformity. Similarly, Damircheli et al. [31] examined the combined effects of temperature and thickness variations on the stress response of rotating FGM disks and confirmed the role of thickness gradients in regulating thermal stress distribution. Their findings showed that thicker intermediate layers can buffer thermal expansion mismatch, while non-uniform thickness distributions contribute to more favorable heat flow paths.

By incorporating thickness optimization into structural design, smoother temperature distributions and heat transfer behavior, closer to ideal continuous gradient models, can be achieved. In practice, thickness optimization is often combined with gradient function optimization to form a multi-parameter synergistic optimization strategy, enabling overall performance enhancement of FGMs.

From a comparative viewpoint, existing studies indicate that gradient function selection, layer discretization, and thickness distribution play complementary but distinct roles in heat transfer optimization of FGMs. Gradient functions primarily regulate the global temperature profile and heat flux distribution, while layer number and thickness optimization mainly affect interfacial thermal resistance and stress localization. Exponential and power-law gradients are typically more effective in reducing peak temperature and thermal stress under large temperature gradients, but their advantages diminish when the gradient is approximated by coarse multilayer discretization. Increasing the layer number improves thermal smoothness but introduces fabrication complexity and diminishing returns beyond a certain threshold. These observations suggest that structural optimization of FGMs should not be treated as independent parameter tuning, but rather as a coupled design problem that balances thermal performance, stress mitigation, and manufacturability.

In summary, existing studies indicate that gradient function selection, layer number, and thickness distribution primarily influence heat transfer performance by modifying effective thermal resistance and temperature gradients across the material. However, it should be noted that conclusions regarding the superiority of specific gradient profiles are often problem-dependent and closely tied to assumptions such as one-dimensional heat flow, steady-state conditions, or simplified boundary conditions. Therefore, gradient optimization strategies should be interpreted as application-specific design guidelines rather than universally optimal solutions.

### 2.2. Topology Optimization Design

Topology optimization is an advanced structural design methodology that aims to achieve specific performance objectives by optimally distributing material and tailoring pore architectures within a defined design domain [32]. In the context of heat transfer design for functionally graded materials (FGMs), topology optimization provides an effective means of generating material configurations with enhanced thermal performance, particularly under large temperature gradient conditions [33]. By employing mathematical optimization algorithms to automatically determine optimal material layouts, topology optimization overcomes many limitations inherent in conventional design approaches.

In the realm of thermal topology optimization, different design objectives and constraints have led to the development of multiple optimization driving mechanisms. Broadly, topology optimization approaches can be categorized into three classes based on their driving features: heat-flux-driven, temperature-field-driven, and thermal-property-driven methods [34].

Heat-flux-driven methods primarily focus on optimizing heat transfer pathways and heat flux distributions, with typical objectives such as minimizing thermal resistance or maximizing average heat flux. These approaches enhance heat transfer efficiency by strategically allocating regions of high and low thermal conductivity to form effective heat conduction channels. Ikonen et al. [35] developed a heat-flux-based topology optimization model for conduction problems and demonstrated that regulating heat flux density distributions can significantly reduce the overall thermal resistance of the system. Under conjugate heat transfer conditions, Meliga et al. [36] employed a level-set-based multi-objective topology optimization framework, achieving a balance between heat flux distribution and convective heat transfer performance.

**Figure 3:**
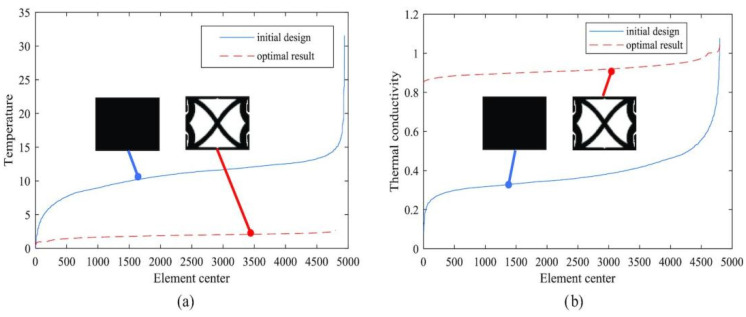
(**a**) temperature distribution of the initial design and the optimal result; (**b**) thermal conductivity of the initial design and the optimal result. Here, “element center” denotes the geometric centroid of each finite element after discretization of the design domain. Reprinted with permission from Ref. [37]. Open access.

Temperature-field-driven methods take temperature uniformity or local temperature constraints as primary objectives, aiming to improve thermal stability by controlling maximum temperature, temperature gradients, or temperature differences. Zhuang et al. [37] proposed a temperature-constrained topology optimization model for nonlinear heat conduction problems, in which the maximum regional temperature was imposed as a constraint. As illustrated in Figure 3, comparisons of temperature distributions and thermal conductivity fields between the initial design and the optimized configuration indicate that the proposed approach effectively mitigates localized overheating. Figure 3 illustrates how temperature-field-driven topology optimization redistributes thermal conductivity to suppress localized overheating, reinforcing the role of temperature-based objectives in controlling heat transfer behavior under nonlinear thermal conditions. These methods are particularly suitable for applications with stringent temperature control requirements, including thermal protection structures, electronic packaging, and phase change thermal energy storage systems.

Thermal-property-driven methods aim to achieve synergistic optimization of heat transfer and structural performance by directly optimizing effective thermal properties, such as thermal conductivity, thermal expansion coefficient, and specific heat capacity. Giraldo-Londoño et al. [38] proposed a multi-material thermomechanical topology optimization framework that directly regulates thermal conductivity distributions at the microstructural level, resulting in significantly improved thermomechanical responses of the optimized structures. Each of these methods exhibits distinct advantages and is suitable for different heat transfer design requirements.

**Figure 4:**
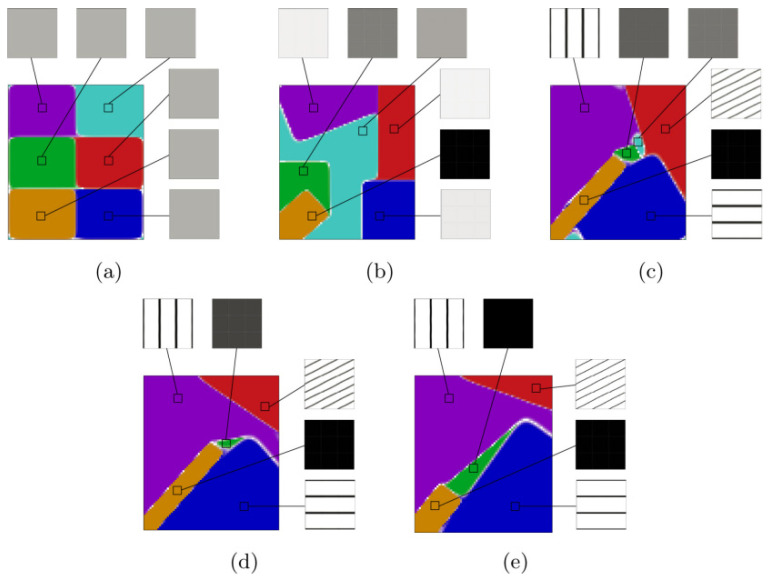
Representative configurations at different iterations of a multi-material topology optimization process for heat transfer design, evolution of micro and macro layouts for five floating patches selected during iteration: (**a**) Iteration 0; (**b**) Iteration 100; (**c**) Iteration 200; (**d**) Iteration 300; (**e**) Iteration 500. Reprinted with permission from Ref. [39]. Copyright ©2019, Elsevier.

Multiscale topology optimization is an important development direction for heat transfer design of FGMs, enabling simultaneous optimization of macroscopic structural layouts and microscopic material architectures. Pizzolato et al. [39] proposed a multiscale, multi-material topology optimization framework that combines level-set and density-based methods, as shown in Figure 4, which illustrates the evolution process of multi-material, multi-scale topology optimization applied to heat sink design. The figure visualizes how macroscopic material subdomains and microscopic material topologies evolve simultaneously during optimization. At early iterations, material subdomains and microstructures are relatively uniform, while at later stages, high-conductivity material progressively migrates and concentrates along dominant heat-conduction pathways, especially near thermally critical regions. This coupled macro–micro evolution highlights how optimized material layouts enhance heat transfer efficiency while limiting the overall usage of high-conductivity material, enabling concurrent design of macro-scale geometry and micro-scale structures. Applications of this framework to heat conduction problems demonstrated that the optimized multiscale structures achieve substantial thermal performance enhancement while maintaining simple macroscopic geometries. The evolution process shown in Figure 4 provides a visual interpretation of how multiscale topology optimization establishes effective heat conduction pathways across macro- and micro-scales, supporting the discussion on hierarchical heat transfer regulation in FGMs.

**Figure 5:**
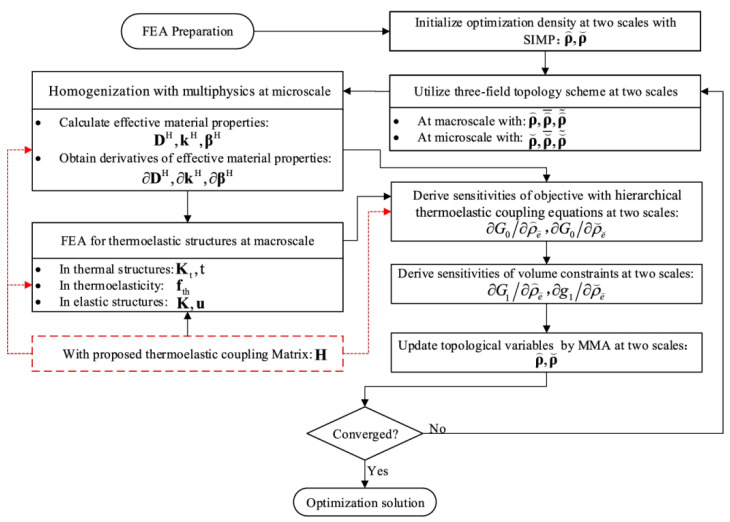
Flow chart of multi-scale concurrent topology optimization method for thermoelastic structures under design temperature field variation. Reprinted with permission from Ref. [41]. Copyright ©2023, Springer Nature.

Da et al. [40] further developed a parallel optimization model that simultaneously designs thermal conductivity distributions at the structural scale and within microscopic multiphase materials, confirming that multiscale collaborative design outperforms single-scale optimization strategies. Guo et al. [41] extended multiscale topology optimization to thermoelastic problems by establishing a coupled optimization model under design-dependent temperature fields. As shown in Figure 5, the optimization framework involves coupled macro-scale and micro-scale variables under thermoelastic loading conditions. At the macro scale, the design variable represents the material distribution within the structural domain, while the temperature field is obtained from the steady-state heat conduction analysis and subsequently used to construct the thermal stress load in the thermoelastic equilibrium equation. The resulting displacement field is then employed to evaluate the mechanical performance objective. At the micro scale, the design variables control the topology of the unit cell, from which the homogenized elastic tensor, effective thermal conductivity tensor, and thermoelastic coupling coefficients are obtained via asymptotic homogenization. These homogenized properties are passed to the macro-scale analysis, enabling a consistent transfer of thermal and mechanical behavior across scales. Sensitivity analysis is performed using adjoint variables associated with the thermal and mechanical governing equations, and the design variables at both scales are updated using the MMA algorithm under prescribed volume constraints and convergence criteria. The optimization workflow, shown in Figure 5, enables effective trade-offs between thermal stress and heat conduction performance, and it clarifies the coupling mechanism between thermal and mechanical fields in multiscale optimization, demonstrating how temperature fields under large gradients directly influence stress redistribution and design updates. In addition, Ali et al. [42] applied multiscale topology optimization to thermal management design of lightweight heat sinks and laser-activated porous actuators, achieving significant heat dissipation enhancement and localized temperature control through microstructural tailoring.

In transient heat transfer analysis, conventional topology optimization methods typically use transient thermal compliance (TTC) as the objective function, defined as the time integral of thermal compliance. Thermal compliance is commonly used as a global performance indicator in heat transfer optimization. It characterizes the overall thermal resistance of a structure and is typically defined as the integral of the temperature field weighted by the applied heat flux, or equivalently as the thermal work conjugate to the heat input. Minimizing thermal compliance corresponds to enhancing the heat dissipation capability of the structure by reducing its overall temperature level under prescribed thermal loading [43,44]. However, this metric does not always fully reflect heat transfer efficiency. To address this limitation, Wu et al. [45] proposed transient thermal dissipation efficiency (TTDE) as a new performance indicator that accounts for the potential dissipation capacity associated with heat absorption induced by temperature rise. Topology optimization models based on TTDE yield designs with higher heat transfer efficiency than those obtained using TTC-based formulations, particularly for transient heat conduction structures requiring rapid thermal dissipation.

**Figure 6:**
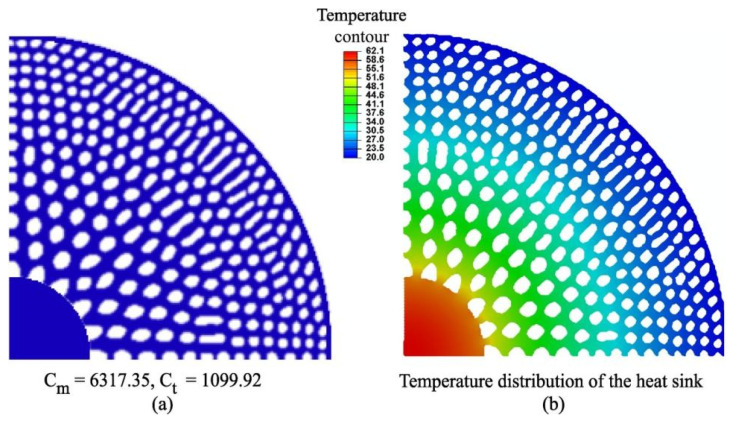
(**a**) Topology optimized graded porous design of radial heat sink with enhanced multi-physics performance, (**b**) Temperature distribution of the radial heat sink. Reprinted with permission from Ref. [46]. Copyright ©2020, Elsevier.

For porous functionally graded materials, topology optimization offers a powerful tool for tailoring microstructures to optimize heat transfer performance. By simultaneously optimizing pore distribution, pore size, and functional gradient parameters at the material scale, synergistic optimization of microstructure and thermal performance can be achieved. Das et al. [46] developed a multiphysics-coupled topology optimization framework for controllable porous FGMs and applied it to thermomechanical optimization of heat-dissipating structures. As shown in Figure 6, C_m_ and C_t_ denote the structural compliance and thermal compliance, respectively. The structural compliance is defined as C_m_ = u^T^K_m_u, where u is the global displacement vector and K_m_ is the global stiffness matrix. The thermal compliance is defined as C_t_ = T^T^K_t_T, where T is the nodal temperature vector, and K_t_ is the global thermal conductivity matrix. These two quantities are commonly used in multiphysics topology optimization to evaluate mechanical stiffness and heat transfer efficiency simultaneously. Figure 6 highlights the necessity of simultaneously considering thermal compliance and structural compliance, illustrating how porous graded designs achieve coordinated optimization of heat dissipation efficiency and mechanical stability. The optimized radially graded porous heat sink design exhibits significantly improved heat dissipation efficiency and structural stability through controlled porosity gradients. Meanwhile, Qureshi et al. [47] constructed porous graded material models based on triply periodic minimal surface (TPMS) unit cells and systematically investigated the effects of porosity and functional gradient distributions on heat transfer during phase change energy storage. Their results demonstrated that graded designs can shorten melting times and enhance heat flux uniformity.

Overall, the heat transfer design of FGMs based on topology optimization can be interpreted from the perspective of thermal resistance redistribution. Heat-flux-driven methods explicitly construct high-conductivity pathways to minimize global thermal resistance, whereas temperature-field-driven methods suppress local hotspots by redistributing material around thermally critical regions. Multiscale topology optimization further extends this concept by enabling coordinated control of heat conduction pathways across length scales. Despite their differences in formulation, these approaches share a common physical objective: reshaping effective heat transfer paths to accommodate large temperature gradients while limiting material usage. Nevertheless, the increased modeling complexity and computational cost of multiscale and multiphysics formulations remain key challenges for practical engineering implementation. From a physical standpoint, thermal topology optimization does not merely rearrange material distribution but fundamentally reshapes heat conduction pathways within the design domain. By concentrating high-conductivity material along dominant heat flux routes and reducing thermal bottlenecks, optimized topologies effectively redistribute thermal resistance and suppress localized temperature peaks, which is particularly critical under large temperature gradient conditions.

### 2.3. Multi-Objective Optimization Design

In the design of functionally graded materials (FGMs), coordinated optimization of thermal and mechanical performance constitutes a complex multi-objective optimization problem [12]. Because material properties in FGMs typically vary continuously along specific directions, designers must seek trade-offs among competing objectives, such as minimizing structural mass while maximizing thermal conductivity, or reducing thermal stresses while maintaining sufficient structural strength. These multi-objective optimization problems usually involve multiple constraints, including stress-based failure criteria, structural response limits, and manufacturing feasibility requirements [48]. Multi-objective optimization strategies frequently integrate both thermal performance and mechanical constraints. Nevertheless, it is essential to recognize that the primary goal of these strategies is thermal optimization, with mechanical factors such as thermal stress incorporated to ensure the structural integrity of FGMs under extreme temperature gradients.

**Figure 7:**
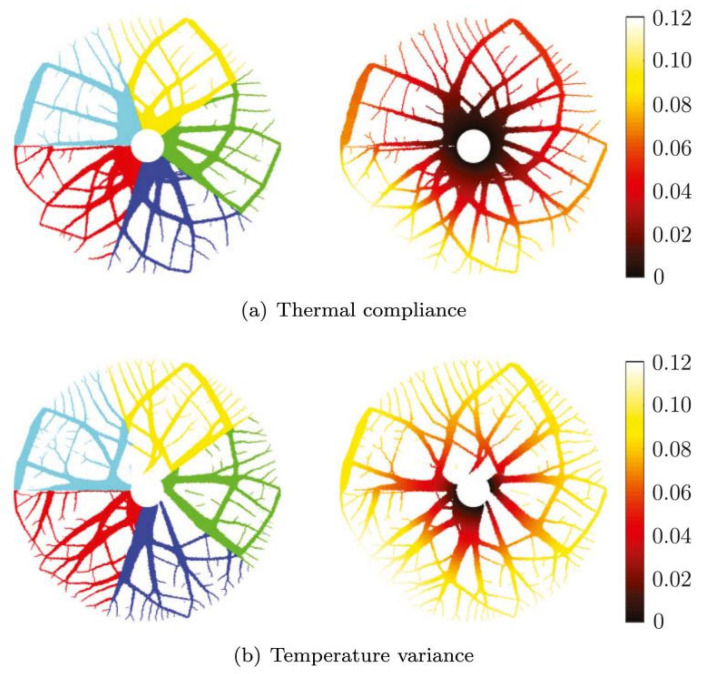
Optimized topologies (**left**) and temperature distribution (**right**) obtained using (**a**) thermal compliance and (**b**) temperature variance. Reprinted with permission from Ref. [38]. Copyright ©2020, Elsevier.

To address the challenges of multi-objective optimization in FGMs, various approaches have been proposed and developed. In parametric optimization, Goupee et al. [12] introduced a multi-objective parameterized design framework incorporating temperature-dependent material properties, in which thermomechanical performance was optimized by adjusting gradient distribution parameters. In the context of intelligent evolutionary algorithms, Correia et al. [49] developed a multi-objective optimization model that simultaneously considers stress-based failure criteria and manufacturing constraints, achieving a balance between structural safety and manufacturability. In multiphysics topology optimization, Das et al. [46] established a coupled optimization framework that simultaneously accounts for heat conduction and mechanical performance, enabling the coordinated optimization of porous FGM structures. Giraldo-Londoño et al. [38] further advanced multi-material thermomechanical topology optimization methods. Figure 7 presents multi-material thermoelastic topology optimization results obtained in an annular (“flower-shaped”) design domain, aiming to investigate the influence of different thermal objective functions on optimized material layouts and temperature responses. Five candidate materials are considered, each subject to an individual regional volume constraint, enabling flexible spatial material allocation. The optimization problem minimizes a weighted combination of mechanical compliance and a thermal objective function, with equal weighting assigned to mechanical and thermal performance. Two different thermal objectives are considered. In Figure 7a, thermal compliance is employed as the thermal objective, promoting a reduction in the overall temperature level of the structure. In contrast, Figure 7b employs temperature variance as the thermal objective, favoring a more uniform temperature distribution across the design domain. Thermal loading is introduced through a uniform volumetric heat generation applied over the entire domain, representing internally generated heat during service. No explicit temperature boundary conditions are prescribed; instead, thermal performance is controlled indirectly through the selected thermal objective functions. From a mechanical perspective, symmetric tangential concentrated loads are applied on the outer boundary, while the inner boundary is fixed, providing a coupled thermo-mechanical loading scenario representative of practical engineering applications. Figure 7 explicitly demonstrates how different thermal objective functions lead to distinct material layouts and temperature responses, underscoring the importance of objective-function selection in multi-objective optimization of FGMs. The topology optimization designs obtained using thermal compliance and temperature variance as objective functions enable optimal material distributions within thermoelastic structures, thereby achieving distinct trade-offs between heat transfer efficiency and temperature uniformity. These studies demonstrate that multiphysics topology optimization provides a powerful framework for integrating thermal and mechanical objectives in FGM design. In surrogate-model-based and intelligent optimization approaches, Nayak et al. [50] combined particle swarm optimization (PSO) with surrogate models for optimizing thermal residual stresses in FGM structures. This hybrid strategy significantly improved computational efficiency and convergence robustness, making it suitable for complex optimization problems with high-dimensional design spaces. From the perspective of manufacturing constraints and process coupling, Li et al. [51] systematically reviewed design criteria for additively manufactured FGMs and emphasized that multi-objective optimization frameworks must explicitly account for gradient continuity, interlayer bonding strength, and manufacturability constraints to ensure practical feasibility.

In recent years, improved multi-objective optimization strategies have been proposed by incorporating decision-variable diversity mechanisms into the optimization process to enhance solution diversity. Shir et al. [52] were among the first to identify insufficient diversity in the decision space of multi-objective algorithms and introduced an adaptive niche radius mechanism in evolutionary processes. By dynamically adjusting local population density in the decision space, this approach actively disperses clustered solutions and improves the uniformity of design variable distributions. Ulrich et al. [53] further developed indicator-based multi-objective evolutionary algorithms (MOEAs) that incorporate diversity measures in both objective space and decision space during fitness evaluation. This dual-space diversity metric framework enables coordinated optimization across both spaces. Pal et al. [54] proposed maintaining diversity simultaneously in objective and decision spaces through decomposition-based multi-objective evolutionary strategies, thereby enhancing global search performance. Segura et al. [55] systematically discussed the importance of decision-space diversity in multi-objective evolutionary algorithms, emphasizing its critical role in avoiding premature convergence. Building on these concepts, Liu et al. [56] proposed a decision-variable-classification-based multi-objective evolutionary algorithm (DVC-MOEA). As shown in Table 1, this framework explicitly incorporates decision-variable diversification mechanisms into the algorithm architecture, significantly enhancing solution set coverage and resolution in complex search spaces. These studies collectively indicate that incorporating decision-variable diversity mechanisms represents an important development direction for multi-objective evolutionary optimization of FGMs.

From an integrative standpoint, studies on multi-objective optimization of FGMs indicate that enhancing heat transfer is rarely pursued as a standalone objective in practical applications. Formulations based on thermal compliance generally prioritize reducing global temperatures, whereas objectives driven by temperature variance focus on achieving thermal uniformity. Hence, the choice of objectives and constraints is highly dependent on the specific application. Evolutionary and surrogate-assisted algorithms are especially effective in managing high-dimensional design spaces and complex constraints; however, their success ultimately relies on the physical relevance of the chosen objectives and design variables. This underscores the importance of aligning optimization formulations with prevailing heat transfer mechanisms and engineering requirements, rather than focusing solely on algorithmic complexity. Collectively, these multi-objective optimization studies demonstrate that no single strategy can maximize all aspects of thermal and mechanical performance simultaneously. Instead, designing FGMs under large temperature gradients necessitates carefully balancing heat dissipation efficiency, temperature uniformity, structural integrity, and manufacturability. This emphasizes the need for problem-oriented optimization frameworks rather than universally applicable design rules.

Although significant progress has been achieved in optimizing heat transfer in functionally graded materials (FGMs), the current literature remains somewhat fragmented. Most studies concentrate on particular optimization strategies, such as topology or multi-objective optimization, often without considering their broader implications in practical applications. For example, although topology optimization has demonstrated potential in improving heat dissipation, the practical limitations imposed by manufacturing constraints are frequently overlooked. Similarly, multi-objective optimization techniques have been widely employed, yet they are often limited in their capacity to effectively address thermo-mechanical coupling in FGMs subjected to large temperature gradients. Furthermore, numerous existing models rely on idealized assumptions that may not fully represent the complexities of actual thermal behavior in FGMs. Regarding optimization algorithms, numerous studies have employed traditional methods, including genetic algorithms and particle swarm optimization, which, although effective, are not always efficient for large-scale or high-dimensional problems. Additionally, most research has predominantly focused on steady-state thermal performance, often neglecting transient heat transfer behavior and long-term material degradation under high thermal loads.

To present a comprehensive overview of optimization strategies for functionally graded materials (FGMs), we summarize a series of commonly employed optimization methods in Table 2. The table highlights key design variables, objective functions, constraints, and engineering applications associated with each method, providing readers with a unified framework to compare and select appropriate strategies for specific heat transfer optimization challenges. Overall, although various gradient functions and geometric configurations have been reported to enhance thermal performance, their effectiveness is highly problem-dependent and closely associated with boundary conditions, heat source distributions, and material nonlinearity. No universally optimal gradient profile can be determined without considering the underlying physical heat transfer mechanisms.

## 3. Numerical Simulation Techniques for Heat Transfer in Functionally Graded Materials

### 3.1. Conventional Numerical Methods

Conventional numerical methods have been well established and widely applied across engineering disciplines [57]. The central idea of these methods is to discretize the continuous solution domain into a finite number of interconnected elements and interpolate field variables using shape functions, thereby transforming partial differential governing equations into a system of algebraic equations suitable for numerical solution [58]. Although challenges arise when applying these methods to nonhomogeneous materials such as functionally graded materials (FGMs), their strong generality and well-established theoretical foundations have made them a cornerstone of FGM performance analysis [59]. While thermomechanical methods are widely employed in FGM studies, this section primarily focuses on heat transfer optimization. Mechanical responses, such as thermal stresses and deformations, are discussed only when they are induced by thermal gradients or when they influence overall heat transfer performance in FGM systems.

**Figure 8:**
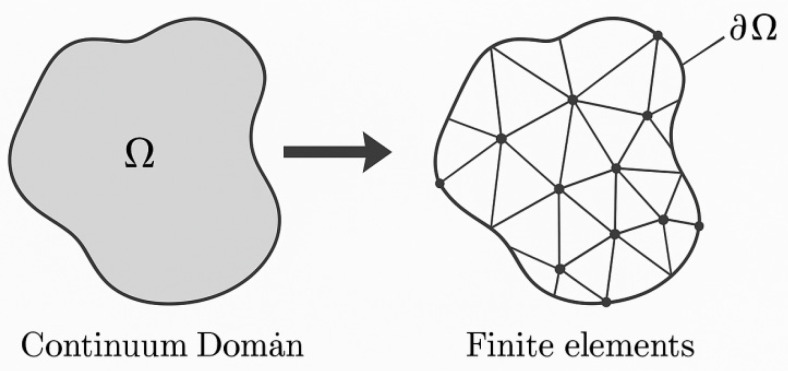
The principle of the finite element method. Reprinted with permission from Ref. [58]. Copyright ©2013, Elsevier.

The finite element method (FEM) is the most widely used conventional numerical method. Its fundamental principle is illustrated schematically in Figure 8, where the continuous domain is discretized into finite elements. When analyzing FGMs, accurately representing spatially varying material properties, such as thermal conductivity and thermal expansion coefficients, is essential. Early approaches, such as that proposed by Vel et al. [60], approximated FGMs as composites consisting of multiple homogeneous layers, with distinct material parameters assigned to each layer to simulate the gradient distribution. While straightforward, this “layer homogenization” approach can introduce significant numerical errors, particularly in regions characterized by steep material gradients or strong temperature-field nonlinearities. To overcome these limitations, Kim et al. [61] developed graded finite element (GFE) techniques that introduce spatial interpolation functions for material properties within individual elements, thereby enabling continuous material variation at the element level and yielding a more accurate representation of physical gradients in FGMs. With advances in computational capabilities, Nguyen-Xuan et al. [62] further combined isogeometric analysis (IGA) with GFE to develop a NURBS-based graded finite element framework that preserves geometric accuracy while achieving high-order approximation of continuously graded materials. These developments have firmly established FEM as a mainstream tool for FGM thermomechanical analysis, encompassing steady-state and transient problems as well as linear and nonlinear regimes. FEM offers significant advantages in handling complex geometries and irregular boundary conditions, while accurately capturing heat conduction and thermally induced stress responses under various coupled loading conditions [63]. Nevertheless, FEM also exhibits notable limitations. Its accuracy depends critically on mesh quality and density; inappropriate meshing can introduce discretization errors and compromise numerical stability [64]. Moreover, in regions with steep property gradients or strong multiphysics coupling, extremely fine meshes are often required to achieve convergence, leading to a substantial increase in computational degrees of freedom and associated time costs [65].

**Figure 9:**
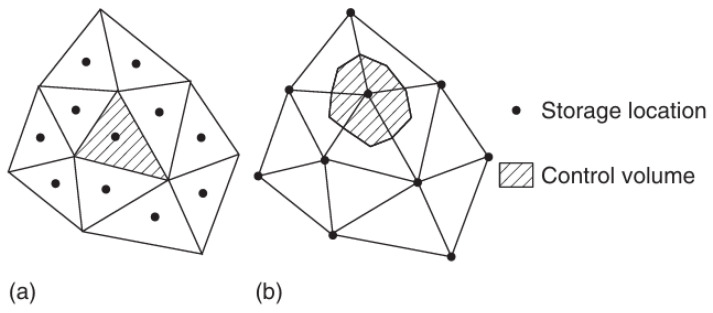
Control volume variants used in the finite volume method: (**a**) cell-centered and (**b**) vertex-centered control volume tessellation. Reprinted with permission from Ref. [66]. Copyright ©2004, John Wiley and Sons.

Alongside FEM, the finite volume method (FVM) represents another major class of conventional numerical approaches. Its fundamental principle is illustrated schematically in Figure 9, which presents two primary strategies for constructing control volumes based on either cell-centered or vertex-based formulations [66]. FVM subdivides the computational domain into non-overlapping control volumes and integrates the governing equations over each volume, thereby ensuring strict conservation of key physical quantities such as mass and energy. This conservation property renders FVM particularly suitable for flow and heat transfer problems characterized by strong gradients or shock phenomena, often exhibiting superior numerical stability relative to FEM [67]. In the context of FGMs, FVM effectively accommodates complex multiphysics processes, including fluid–solid interactions, enabling accurate resolution of internal thermal responses.

Charoensuk et al. [68] proposed a high-order control-volume finite element method (CVFEM). As summarized in Table 3, predictive quality is employed to evaluate the accuracy of numerical heat conduction models, including the conventional FEM and CVFEM, through comparisons between predicted temperature fields and corresponding analytical solutions. Predictive quality is quantified by the relative error between numerical and exact temperature values, with smaller errors indicating higher prediction accuracy. The results demonstrate that CVFEM yields superior accuracy relative to conventional FEM, indicating that the incorporation of higher-order interpolation functions within hybrid meshes significantly enhances simulation precision for transient heat conduction in multilayer FGMs. Gong et al. [69] further proposed an unstructured finite volume time-domain method (UFVTDM) aimed at improving computational stability and geometric adaptability in multilayer FGM simulations. Despite these advantages, FVM shares several limitations with FEM. Its accuracy remains strongly dependent on mesh quality, and mesh generation for complex geometries can be computationally cumbersome [70]. Moreover, in regions exhibiting steep material property gradients, high-density meshes are required to maintain solution accuracy, leading to substantially increased computational costs and time consumption [71].

### 3.2. Emerging Numerical Methods

To overcome the inherent limitations of conventional mesh-dependent methods, a range of emerging numerical techniques has been developed in recent years, exhibiting strong potential for handling steep gradients, large deformations, and discontinuities in functionally graded materials (FGMs) [72]. A key advancement of these methods lies in either eliminating dependence on traditional meshes or enabling seamless integration between geometric and analysis models, thereby providing new paradigms for FGM performance evaluation.

**Figure 10:**
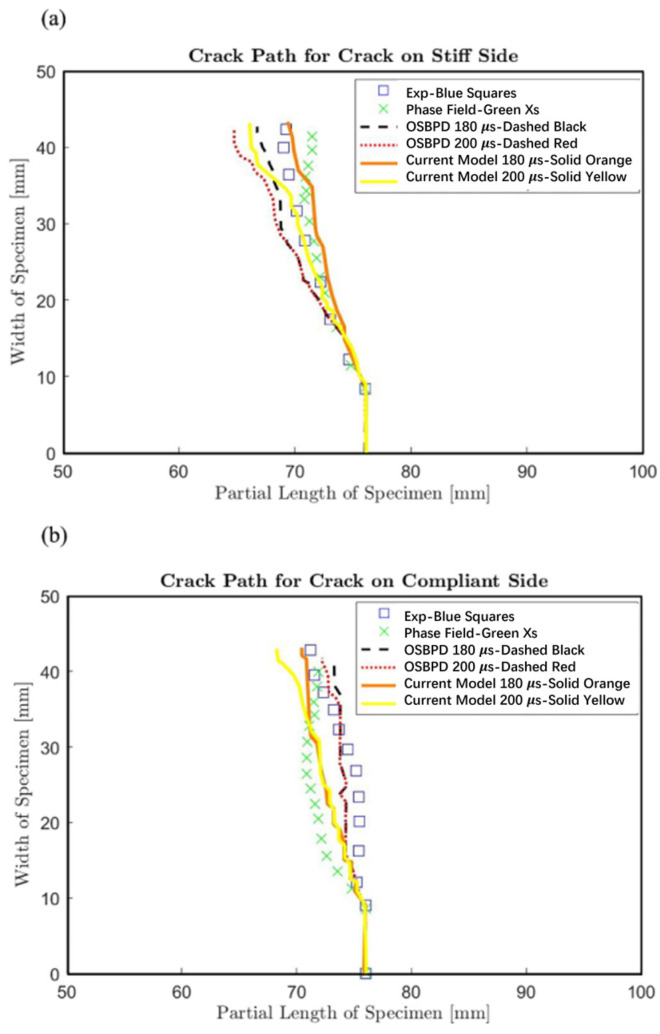
Results obtained based on Timoshenko’s MPPD model and comparison of crack paths with experimental and other numerical results: (**a**) cracks on the harder side; (**b**) cracks on the softer side. Reprinted with permission from Ref. [74]. Copyright ©2025, Wiley Online Library.

Peridynamics (PD) is a mesh-free, nonlocal theoretical framework in which the constitutive relations and equations of motion of a continuum are formulated in an integro-differential form. In PD, the material domain is discretized into interacting particles, allowing macroscopic behavior to emerge from particle-level interactions. Unlike traditional numerical methods, PD does not require a predefined mesh; instead, the computational domain is characterized by the spatial distribution of particles, thereby inherently avoiding mesh distortion and boundary layer errors [73]. This feature renders PD particularly suitable for simulating transient thermal responses and heat conduction in FGMs, especially for problems involving crack propagation and other discontinuities. Bautista et al. [74] integrated PD with FGM analysis to develop a crack propagation model based on multipole Timoshenko peridynamics (MPPD), as illustrated in Figure 10, thereby demonstrating the advantages of PD in addressing discontinuities in FGMs. Despite these strengths, PD remains computationally demanding, particularly for large-scale particle systems [75]. Furthermore, the accuracy of PD models is highly sensitive to the selection of influence functions and interaction lengths, necessitating careful parameter calibration [76].

Isogeometric analysis (IGA) represents a complementary numerical approach that emphasizes geometric exactness. Its core idea is to unify computer-aided design (CAD) and computer-aided engineering (CAE) by directly employing CAD basis functions (e.g., non-uniform rational B-splines, NURBS) as shape functions within finite element analysis. This strategy thereby enables accurate geometric representation while avoiding the approximation errors inherent in conventional FEM mesh generation [77]. Hughes et al. [78] demonstrated that IGA achieves significantly higher accuracy than conventional FEM for thermal conduction problems in functionally graded materials (FGMs), even with fewer degrees of freedom, which constitutes a critical advantage for thermoelastic and thermal buckling analyses. Cottrell et al. [78] further emphasized that IGA not only eliminates traditional geometry-to-analysis conversion errors but also maintains high geometric fidelity throughout the computational process. Moreover, IGA enables higher-order continuity, which is particularly important for accurately capturing stress and temperature gradients in FGMs. Nevertheless, IGA faces several challenges: converting CAD models into NURBS-compatible representations requires substantial preprocessing effort [79], and complex geometries composed of multiple NURBS patches remain cumbersome to manage, thereby limiting their widespread adoption in engineering practice [80].

**Figure 11:**
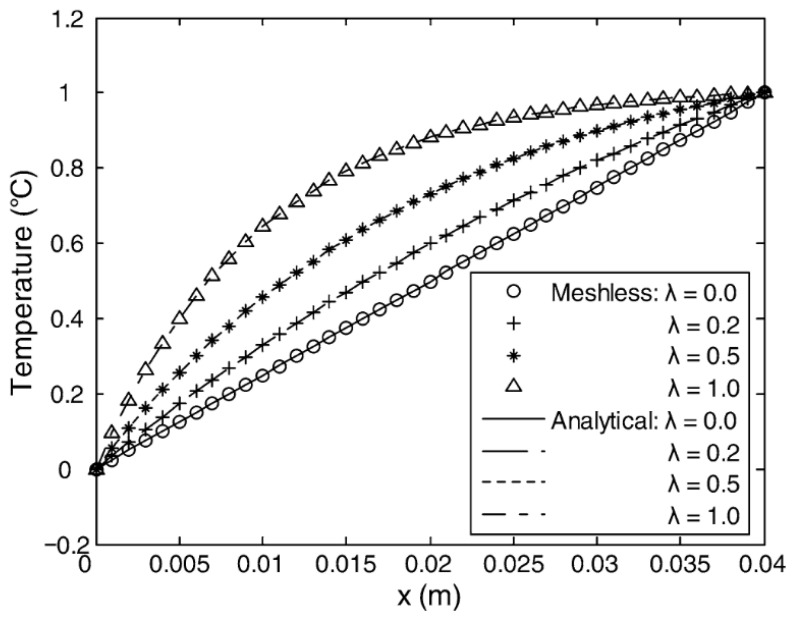
Distribution of temperature along the x-axis for a functionally graded finite square strip under steady-state loading conditions. Reprinted with permission from Ref. [84]. Copyright ©2006, Springer Nature.

Meshfree methods (MFMs) constitute a broad class of numerical techniques characterized by their independence from conventional meshes. In these methods, field variables are approximated using nodes or particles with shape functions defined over their respective influence domains, such as radial basis functions. Belytschko et al. [81] systematically introduced the element-free Galerkin (EFG) method, thereby establishing a solid theoretical foundation for meshfree computation. Compared with peridynamics (PD), MFM approaches are more closely aligned with classical continuum mechanics and are therefore more readily interpreted by engineers. Liu et al. [82] highlighted that MFM simplifies the modeling of complex geometries and is well-suited for problems involving large deformations and intricate boundary conditions. In FGMs, MFM has been successfully applied to analyses of thermoelastic behavior, transient heat conduction, and coupled rigid–flexible dynamic systems. Atluri et al. [83] demonstrated that the meshless local Petrov–Galerkin (MLPG) method offers notable computational advantages for complex physical problems. Wang et al. [84] further applied MFM to transient heat conduction in FGMs, obtaining high-accuracy solutions consistent with analytical predictions. Figure 11 illustrates a representative temperature distribution along the x-axis of a functionally graded finite square strip under steady-state conditions, demonstrating excellent agreement between numerical and analytical results. However, similar to PD, the computational cost of MFM increases with the number of nodes, and certain approaches—such as those based on radial basis functions—may suffer from ill-conditioning and reduced numerical stability [85].

### 3.3. Multiphysics Coupled Analysis

**Figure 12:**
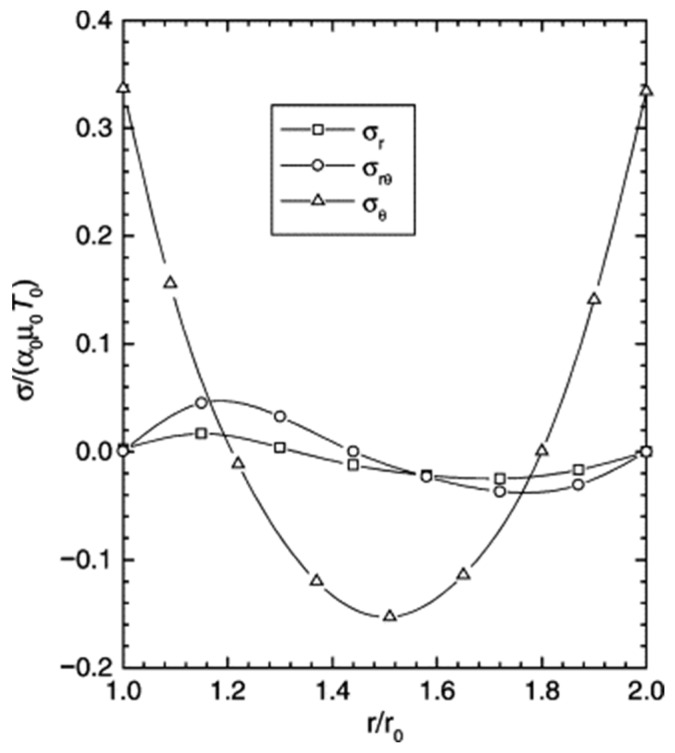
Thermal stresses induced by θ-dependent temperature (*n* = 2, *q* = 0). Reprinted with permission from Ref. [90]. Copyright ©2003, Elsevier.

In practical engineering applications, particularly in aerospace and energy systems, the performance of functionally graded materials (FGMs) is almost invariably governed by interactions among multiple physical fields, including thermal, mechanical, electrical, and magnetic phenomena. To address these complexities, multiphysics coupled analysis frameworks have been developed and widely applied in FGM modeling. In this context, it is important to clarify that many studies reviewed in this subsection primarily focus on mechanical responses—such as thermal stress, buckling, or crack propagation—while temperature fields and heat transfer behavior act as the underlying driving mechanisms rather than the primary optimization objectives. Suresh et al. [86] systematically summarized the fabrication processes and thermomechanical responses of FGMs, emphasizing that compositional gradients directly affect thermal expansion behavior and stress distributions, thereby necessitating the simultaneous consideration of thermal and mechanical fields in material and structural design. Reddy et al. [87] conducted thermomechanical analyses of cylindrical and plate FGMs, demonstrating that high-temperature gradients significantly alter stress fields and influence structural instability and fracture characteristics. Reddy [88] further developed a higher-order finite element model for FGM plates subjected to thermal loading, numerically illustrating variations in buckling behavior and stress distributions, and thereby indirectly highlighting the critical role of temperature distribution and heat transfer behavior in determining FGM performance. Birman et al. [89] reviewed numerous engineering case studies, concluding that single-physics analyses are insufficient for accurately predicting the performance of components such as turbine blades, disks, and electronic heat sinks. Empirical investigations further indicate that temperature fields strongly influence thermal conductivity, material strength, and fracture toughness, thereby affecting the mechanical and electromagnetic responses of components operating under high-temperature gradients. Liew et al. [90] analyzed the thermal stress behavior of cylindrical and plate FGMs under thermal loading conditions, as shown in Figure 12, demonstrating the essential role of thermomechanical coupling in engineering components such as blades and cylindrical structures.

Therefore, a comprehensive and reliable prediction of FGM performance must go beyond single-field analyses and adopt multiphysics coupled approaches. Only through the integrated consideration of thermal, mechanical, electrical, and magnetic field interactions can accurate simulation and prediction of FGM behavior under complex service conditions be realized.

## 4. Heat Transfer Optimization of Functionally Graded Materials in Engineering Applications

From an application-oriented perspective, heat transfer optimization of functionally graded materials (FGMs) is commonly achieved through a combination of geometric parameter optimization, material gradient tailoring, topology optimization, and, in some cases, multi-objective optimization frameworks that account for coupled thermomechanical responses. In engineering practice, these optimization strategies are typically integrated into system-level design processes rather than treated as standalone numerical algorithms. Accordingly, this section focuses on how different optimization approaches are practically implemented in representative engineering scenarios, with emphasis on their design objectives, optimization variables, and associated thermal performance improvements. To provide a clearer understanding of the underlying techniques, this section further examines the specific optimization strategies employed in the design process.

In the context of heat transfer optimization, several methods have proven effective in enhancing thermal performance under large temperature gradients. One key technique is topology optimization, which seeks to optimize material distribution within a prescribed design domain. By adjusting the shape and configuration of the material layout, topology optimization can effectively reduce thermal resistance and promote efficient heat conduction pathways. For instance, in the design of heat sinks and thermal barrier coatings, topology optimization has been demonstrated to enhance heat dissipation while simultaneously minimizing component weight and material usage.

Another important approach is structural optimization, which focuses on optimizing geometric configurations and material properties of FGMs to improve their heat transfer characteristics. This strategy accounts for factors such as temperature gradients, material heterogeneity, and thermally induced stresses, with the objective of balancing structural integrity and thermal conductivity. Gradient profiles, including linear and exponential distributions, are frequently optimized to mitigate thermal stress concentrations and enhance overall heat flow efficiency within the material.

Multi-objective optimization has emerged as a critical strategy, particularly for FGM designs that must simultaneously satisfy multiple performance criteria, such as thermal efficiency, structural strength, and manufacturability. This approach addresses competing objectives by concurrently optimizing material properties and layout configurations. Algorithms such as genetic algorithms, particle swarm optimization (PSO), and other evolutionary techniques are widely employed to identify optimal solutions that meet heat dissipation requirements while maintaining structural reliability and complying with manufacturing constraints.

Each of these optimization techniques targets specific challenges associated with large temperature gradients and nonlinear heat transfer behavior. By carefully selecting appropriate methods and adapting them to application-specific requirements, optimal heat transfer performance can be achieved, thereby ensuring that FGM designs are both thermally efficient and mechanically robust.

### 4.1. Applications in Electronic Thermal Management Systems

With the continuing trend toward miniaturization and increasing power density of electronic devices, heat dissipation has become a critical factor constraining the performance and reliability of modern electronic systems. Functionally graded materials (FGMs), owing to their tunable thermophysical properties and adaptable structural characteristics, have demonstrated significant potential for application in electronic thermal management systems. To address these thermal management challenges, researchers have explored the integration of phase change materials (PCMs) with functionally graded structures to achieve efficient heat dissipation under multiphysics operating conditions. Hoe et al. [91] proposed a target-oriented optimization framework for PCM-based composite heat sinks, in which phase change materials were embedded within high-thermal-conductivity substrates. Through geometric optimization, thermal resistance was significantly reduced, thereby effectively suppressing peak device temperatures. Arshad et al. [92] investigated the thermal performance of PCM-based heat sinks incorporating nanoparticles and metal foams. Their results showed that the inclusion of nanoparticles and metal foams significantly enhances thermal conductivity and latent heat storage capacity of PCMs, thereby improving overall thermal management performance and providing valuable insights for the design of more efficient electronic cooling systems.

Regarding the integration of thermal energy storage and heat dissipation, Ye et al. [93] pointed out that although latent heat energy storage materials theoretically exhibit high energy densities, the system-level energy density is often substantially lower due to limitations related to heat conduction, structural packaging, and system integration. This observation indicates that, for volume-constrained electronic cooling systems, comprehensive design and optimization must be conducted from a system-level perspective. Meanwhile, Zou et al. [94] investigated an integrated electronic cooling and energy harvesting system based on ferroelectric polymers, revealing synergistic thermo–electric coupling effects on temperature regulation and energy harvesting performance. Their work provides strong theoretical and experimental support for the development of novel self-powered electronic systems.

At the nanoscale, piezoelectric polarization effects induced by non-uniform temperature fields have emerged as an active research focus. Chen et al. [95], based on a functionally graded flexible piezoelectric beam model, analyzed polarization responses induced by thermal strain gradients and demonstrated that such effects enable thermoelectric energy conversion in micro- and nanoscale structures. This finding opens new research avenues for the design of FGM-based heat dissipation systems with integrated self-sensing and energy harvesting functionalities under extreme service conditions, such as high-gravity environments or large temperature gradients.

Overall, optimization strategies in electronic thermal management applications are primarily implemented through geometric optimization of heat sinks, tailored material distribution within FGMs, and system-level integration of phase change, thermoelectric, or ferroelectric components to minimize peak temperature and thermal resistance. From a physical perspective, these optimization approaches focus on redistributing heat flow and reducing thermal resistance. Functionally graded materials enable efficient heat conduction pathways that facilitate heat dissipation away from localized hot spots, while simultaneously alleviating stress concentrations arising from thermal expansion mismatches. These mechanisms are fundamental to enhancing the longevity and reliability of electronic components operating under high thermal loads.

Looking ahead, the development of FGMs in electronic thermal management systems is expected to progress toward multifunctional and intelligent solutions. By coupling ferroelectric, thermoelectric, and phase change materials through rational multiphysics design strategies, integrated intelligent cooling systems that combine heat dissipation, energy harvesting, and sensing capabilities can be realized. Furthermore, with continued advances in multiphysics numerical simulation techniques and optimization algorithms, the precise design and performance prediction of FGMs will become increasingly efficient and reliable, providing robust support for the thermal management of next-generation high-power electronic devices.

### 4.2. Applications in the Aerospace Field

Functionally graded materials (FGMs), characterized by continuous variations in composition and structure, play a crucial role in aerospace thermal protection systems. Suresh et al. [86] systematically reviewed fabrication techniques and thermomechanical behavior of FGMs, demonstrating that gradient-based designs can significantly improve thermal stress distributions and enhance thermal resistance. Consequently, FGMs are particularly well suited to satisfying thermal protection requirements under extreme service conditions encountered during flight, where temperatures may span a wide range. Birman et al. [89] offered a comprehensive review of FGM modeling and engineering applications, covering numerous studies related to heat conduction, thermomechanical coupling, instability, and fracture. They emphasized that analytical approaches incorporating material non-homogeneity and multiphysics coupling effects are essential for the design of aerospace thermal protection systems.

**Figure 13:**
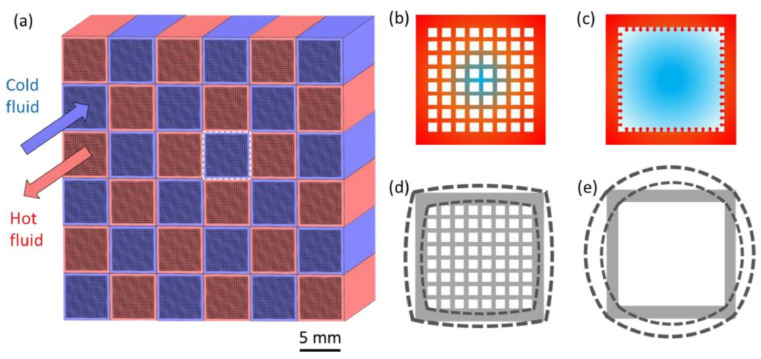
Schematics of the multiscale porous ceramic heat exchanger design. (**a**) The counterflow heat exchanger consists of macrochannels (white dashed box) in a checker-board pattern. (**b**) A single macrochannel comprises the microchannel array, enhancing the heat spread in the macrochannel due to lower thermal resistance of the thin solid walls, compared to the conventional macrochannel in (**c**). (**d**) The microchannel walls also reinforce the mechanical strength. (**e**) Comparably without microchannels, the macrochannel walls experience significantly higher stress and deformation. The dashed lines in (**d**,**e**) represent the magnified deformation of the macrochannel walls under the high-pressure loadings. Reprinted with permission from Ref. [96]. Copyright ©2022, Elsevier.

In the field of high-temperature heat exchangers, multiscale porous ceramic heat exchangers represent an emerging frontier technology in aerospace thermal management. Although conventional high-temperature heat exchanger designs can withstand high-temperature and high-pressure environments, such designs are often associated with high manufacturing costs and relatively low power density. In this context, Li et al. [96] proposed and numerically validated a multiscale porous ceramic heat exchanger, as schematically illustrated in Figure 13. The results demonstrated that optimization of multiscale macroscopic and microscopic channel structures can significantly enhance heat transfer efficiency and power density while maintaining an extremely low pressure drop. This design offers a scalable and efficient thermal management solution for propulsion systems and high-temperature power devices.

Owing to their continuously varying material composition and thermophysical properties from the surface to the interior, functionally graded materials (FGMs) offer significant advantages in the design of thermal barrier coatings (TBCs), structural component protection, and heat conduction and dissipation systems. Lee et al. [97] first introduced the concept of metal–ceramic composite materials for thermal barrier coating applications, highlighting that varying compositions during spraying can reduce thermal stresses at interfaces caused by expansion coefficient mismatches, thus improving high-temperature reliability. Subsequently, Boggarapu et al. [98] summarized thermal conduction analyses, homogenization models, and gradient-based structure designs of FGMs, emphasizing the critical role of gradient distribution and heat flow orientation in reducing thermal gradients and stresses.

Various strategies have been employed to optimize heat transfer performance. In particular, Şafak et al. [99] conducted a parametric sensitivity analysis, showing that an increase in the gradient index β enhances both the heat dissipation rate and fin efficiency of FGM annular fins. Additionally, designs featuring non-uniform material distributions exhibit reduced temperature gradients and enhanced heat transfer compared to homogeneous fins. Multi-objective optimization methods have been utilized to balance thermal performance, structural mass, and cost. Wang et al. [100] applied a micro-genetic algorithm to optimize composition distributions of thermal barrier coatings (TBCs) or functionally graded coatings. Their results demonstrated that optimized composition distributions reduce thermal risks under heat flux loading, lower peak thermal stresses, and extend material lifespan under high-temperature conditions.

In aerospace applications, heat transfer optimization of FGMs primarily relies on gradient index design, parametric sensitivity analysis, and multi-objective optimization strategies that balance thermal performance, structural integrity, and material costs in extreme thermal environments. In aerospace thermal protection systems, FGMs are optimized to create graded thermal pathways that direct heat away from critical components. This not only improves heat dissipation but also effectively mitigates thermal stress concentrations, which can lead to material failure. The physical basis for this optimization lies in the redistribution of thermal resistance, essential for maintaining structural integrity under high-temperature gradients. Looking ahead, the application of functionally graded materials in aerospace thermal protection systems holds substantial development potential. With advancements in computational simulation technologies, multiscale modeling and optimization techniques are expected to further improve FGM performance. Furthermore, the development of intelligent thermal management systems will enable FGMs to autonomously adjust their thermal properties based on flight conditions, further enhancing the safety and efficiency of aerospace vehicles.

### 4.3. Applications in Energy Systems and Building Energy Conservation

In the fields of energy systems and building energy conservation, functionally graded materials (FGMs) exhibit substantial potential for application due to their unique thermophysical properties and design flexibility. By optimizing the spatial distribution of parameters such as thermal conductivity and specific heat capacity, FGMs can significantly enhance energy conversion efficiency and improve indoor thermal comfort, offering innovative solutions for energy savings and emission reductions. As renewable energy technologies and energy-efficient buildings evolve, FGMs have found increasing application in optimizing heat transfer performance in energy systems and building energy conservation. FGMs facilitate continuous variation in material composition along thickness or spatial directions, thereby regulating thermophysical parameters such as thermal conductivity and thermal expansion coefficients, enabling precise control of heat flow.

Tong et al. [101] proposed integrating functionally graded structures with topology optimization for cooling plate design. The results indicated that the adoption of a graded lattice structure significantly reduced the temperature difference and peak temperature of the cooling plate, compared to conventional homogeneous structures, thereby improving temperature uniformity and thermal management performance. FGM transition layers were also employed by Dose et al. [25] to mitigate thermal stresses in plasma-facing components of nuclear fusion systems. Adjusting the alloy concentration gradient within the layer mitigated thermal strain mismatch, resulting in enhanced long-term reliability.

FGMs have been initially applied to exterior walls and curtain wall panels in building envelope structures to enhance energy efficiency. Chen et al. [102] studied aluminum–polymer FGM panels in photovoltaic–thermal (PV/T) combined systems. By transferring heat generated in photovoltaic cells to water pipes via graded materials, the operating temperature of the cells was reduced, and thermal energy was recovered. Compared to panels made of purely metallic or polymeric materials, this design demonstrated significantly reduced interfacial temperature differences and heat losses. Additionally, to address linear heat sources or localized thermal loading in building envelope structures, Tian et al. [18] analyzed the impact of material composition gradient parameters along the thickness direction on temperature distribution using a hybrid numerical method. The results showed that with a large gradient index, the temperature near the heat source increased, while the overall temperature gradient was mitigated, making such designs effective for suppressing thermal shock in walls or roofs.

In energy systems and building applications, optimization is typically achieved by tailoring through-thickness material gradients and utilizing topology or lattice optimization to enhance temperature uniformity, reduce thermal stress, and improve overall energy efficiency. In energy systems and building energy conservation, the principle of optimizing thermal resistance distribution ensures that heat transfer is maximized in critical areas and minimized in less critical regions, thereby balancing energy efficiency with structural integrity, particularly under extreme conditions such as high temperature gradients.

With ongoing advances in materials science and energy technologies, the application of functionally graded materials in energy systems and building energy conservation is anticipated to become increasingly widespread. On one hand, the development of novel FGMs is expected to further enhance energy conversion efficiency and building thermal performance. On the other hand, integrating intelligent control technologies with FGMs will enable precise regulation and optimized operation of energy systems. In multi-energy complementary systems and regional energy networks, FGMs are anticipated to become a crucial technology for linking different energy forms and optimizing energy distribution, thereby supporting the development of low-carbon, efficient, and sustainable energy systems.

It is important to note that the apparent superiority of certain optimization algorithms reported in the literature is often closely linked to the selected design variables, objective functions, and physical assumptions. In most cases, optimization outcomes reflect how effectively a given method redistributes thermal resistance, smooths temperature gradients, or controls heat flux pathways, rather than highlighting an intrinsic advantage of the algorithm itself.

## 5. Conclusions and Perspectives

As a critical link between materials science and engineering applications, functionally graded materials (FGMs), owing to their continuously tunable composition and properties, demonstrate exceptional thermal stability and heat transfer regulation capabilities under large temperature gradient conditions. This review provides a systematic summary of recent research advancements in heat transfer design optimization for FGMs. Heat transfer optimization studies of FGMs, spanning structural and compositional optimization, topology optimization, and multi-objective optimization methods, in key application fields—such as electronic thermal management, aerospace, energy systems, and building energy conservation—are comprehensively reviewed and analyzed. Overall, FGMs have emerged as a significant class of materials for addressing complex thermal management challenges across various fields.

From a state-of-the-art perspective, current research on heat transfer optimization of FGMs has largely shifted from parametric studies to topology-based and multiphysics-driven design frameworks. However, many reported optimization outcomes remain heavily dependent on simplified physical models and problem-specific assumptions. A major challenge lies in bridging the gap between numerical optimization results and physically interpretable design principles that can be generalized to different thermal loading scenarios.

Large temperature gradients in functionally graded materials (FGMs) typically induce significant nonlinearity in heat transfer behavior, as well as notable temperature-dependent changes in material properties such as thermal conductivity and expansion. These effects are critical for understanding the thermal performance of FGMs in high-power, high-temperature environments and must be carefully considered in optimization studies. Thus, future work in heat transfer optimization for FGMs must account for the complex interplay between thermal stresses, material behavior, and heat conduction under large temperature gradients.

Despite substantial progress in numerical simulation methods, material fabrication technologies, and multiphysics-coupled analyses in recent years, heat transfer optimization of FGMs continues to face several challenges. First, many existing models rely on idealized assumptions, and the representation of interfacial defects, microscopic heterogeneity, and temperature-dependent material properties remains inadequate. Second, conventional optimization designs often prioritize single objectives, and there remains a need to establish multi-objective optimization frameworks that simultaneously consider heat transfer performance, structural strength, and manufacturing feasibility. Furthermore, comprehensive evaluation systems for the service reliability of FGMs under high-temperature, strong radiation, and complex thermo-mechanical environments have yet to be fully developed.

In conclusion, although the current state of the art in heat transfer optimization of FGMs has made notable progress, much work remains to be accomplished. Existing research predominantly addresses isolated aspects of thermal performance, such as heat dissipation and material distribution, without adequately considering the complexities of multiphysics interactions and real-world constraints. Furthermore, there is a lack of consensus on the most appropriate optimization approaches for various application scenarios, particularly under large temperature gradients.

Future research should prioritize the development of more integrated optimization frameworks that simultaneously consider both thermal and mechanical performance of FGMs. These frameworks should also address challenges posed by complex manufacturing processes, ensuring that optimized designs are both theoretically robust and practically feasible. Additionally, advancements in computational methods and multiscale modeling will be critical in capturing the complex behaviors of FGMs under extreme thermal loads. Only through these efforts can truly optimized FGM designs be achieved, offering superior performance in practical applications.

Looking forward, heat transfer optimization of FGMs is anticipated to evolve towards intelligent, multiscale, and cross-disciplinary coupling. Multiscale modeling and simulation are expected to become essential tools for elucidating the relationships between gradient structures and thermal performance. The incorporation of machine learning and data-driven optimization methods is expected to enable a paradigm shift from “design-driven” approaches to “performance-inverse design.” Regarding manufacturing technologies, the integration of additive manufacturing and graded coating techniques will substantially enhance the controllability and precision of functionally graded structures. Simultaneously, multifunctional graded systems incorporating phase change materials, thermoelectric materials, and ferroelectric materials will pave the way for integrated heat dissipation, energy harvesting, and intelligent sensing.

Ultimately, FGMs are anticipated to play an increasingly pivotal role in high-temperature thermal protection, microelectronic thermal management, and sustainable buildings and energy systems, thereby continually advancing heat transfer engineering toward higher efficiency, lower carbon emissions, and greater intelligence.

## Figures and Tables

**Table 1:** The main framework of DVC-MOEA. Reprinted with permission from Ref. [56]. Copyright ©2022, Elsevier.

1: [DV,CV,Pro,Interval] = DVC; /** Algorithm 1 **/
2: *P* ← Initialization (CV,Pro,Interval);
3: *CA* ← CA Optimization (Ø, *P*); // convergence archive
4: *DA* ← DA Optimization (Ø, *CA*); // diversity archive
5: **while** termination criterion not fulfilled **do**
6: *Q* ← Offspring Creation by DC (*CA*, *DA*, *DV*, *CV*); /** Algorithm 6 **/
7: *CA* ← CA Optimization (*CA*, [*P*, *Q*]);
8: *DA* ← DA Optimization (*DA*, *CA*);
9: *P* ← Environmental Selection ([*P*, *Q*]);
10: **end while**
11: **return** *P*;

Notation: *DV* denotes the diversity-related decision variables that mainly influence the distribution of solutions along the Pareto front; *CV* represents the convergence-related decision variables that control the convergence speed toward the Pareto-optimal set; *P* is the parent population; *Q* is the offspring population generated during evolutionary operations; *CA* refers to the convergence archive, which stores solutions with superior convergence performance; *DA* denotes the diversity archive, which maintains a well-distributed set of solutions in the objective space.

**Table 2:** Comparison of optimization methods.

Optimization Method	Design Variables	Objective Function(s)	Constraints	Engineering Applications
Structural Optimization	Material distribution, geometry	Minimize thermal compliance, maximize heat dissipation	Volume fraction, manufacturability	Electronic cooling systems, heat sinks
Topology Optimization	Element density, material layout	Minimize thermal resistance, maximize heat dissipation	Stress constraints, material distribution limits	Aerospace thermal protection, high-temperature devices
Multi-objective Optimization	Material gradients, design geometry	Maximize heat dissipation, structural strength	Volume constraints, temperature limitations	Aerospace, energy systems, electronic devices
Phase Change Materials Integration	Phase change material distribution, material gradients	Maximize thermal storage and dissipation	Temperature stability, phase transition constraints	Electronic devices, thermal storage systems
Gradient-based Optimization	Thermal conductivity, material gradients	Minimize temperature variance, maximize heat transfer efficiency	Geometric constraints, material limits	Porous fins, energy-efficient buildings
Parametric Sensitivity Analysis	Geometric parameters, material distribution	Maximize heat dissipation efficiency, optimize structural integrity	Thermal flux, material distribution	Nuclear fusion systems, high-performance electronics

**Table 3:** Comparison of predictive quality between conventional FEM and CVFEM. Reprinted with permission from Ref. [68]. Copyright ©2010, Springer Nature.

*λ*	*x*	Exact	FEM (6 Node)		CVFEM (6 Node)	
			*T*_FEM_	Error (%)	*T*_CVFEM_	Error (%)
0.2	0.01	0.329179	0.309017	6.12489	0.32932	0.043493
	0.02	0.598688	0.599746	0.17678	0.59868	0.000611
	0.03	0.819343	0.805994	1.62921	0.81944	0.011738
0.5	0.01	0.455054	0.394336	13.3431	0.45772	0.585373
	0.02	0.731059	0.733122	0.28225	0.73101	0.006371
	0.03	0.898464	0.87677	2.41453	0.89944	0.108555

Notes: *λ* is the material non-homogeneity parameter. *x* is the spatial coordinate along the heat transfer direction (m). Exact denotes the analytical temperature solution. *T*_FEM_ and *T*_CVFEM_ represent the temperature values obtained by the conventional FEM and the CVFEM, respectively. The relative error is defined as |T_numerical_ − T_Exact_|/T_Exact_ × 100%.

## Data Availability

No new data were created or analyzed in this study. Data sharing is not applicable to this article.

## References

[1] Incropera F.P., DeWitt D.P., Bergman T.L., Lavine A.S. (2011). Fundamentals of Heat and Mass Transfer.

[2] Lagow B.W. (2016). Materials Selection in Gas Turbine Engine Design and the Role of Low Thermal Expansion Materials. JOM.

[3] Umana E.M., Yang X. (2025). Review of Film Cooling Techniques for Aerospace Vehicles. Energies.

[4] Gilbey D.M. (1959). Theory of thermal stresses. J. Less Common Met..

[5] Zhao J., Qiu F., Xu C. (2023). Review of Creep-Thermomechanical Fatigue Behavior of Austenitic Stainless Steel. Crystals.

[6] Wang R., Liao D., Zhang X., Zhu S., Tu S., Guo S. (2021). Creep-fatigue Life Design Methods in High-temperature Structures: From Materials to Components. J. Mech. Eng..

[7] Bohidar S., Sharma R., Mishra P. (2014). Functionally graded materials: A critical review. Int. J. Res..

[8] Fuchiyama T., Noda N. (1995). Analysis of thermal stress in a plate of functionally gradient material. JSAE Rev..

[9] Sasaki M., Hirai T. (1991). Fabrication and properties of functionally gradient materials. J. Ceram. Soc. Jpn..

[10] Ji Q., Huang J.-P. (2018). Controlling thermal conduction by graded materials. Commun. Theor. Phys..

[11] Zhang T., Zhang K., Liu F., Zhao M., Zhang D.Z. (2024). Analysis of thermal storage behavior of composite phase change materials embedded with gradient-designed TPMS thermal conductivity enhancers: A numerical and experimental study. Appl. Energy.

[12] Goupee A.J., Vel S.S. (2007). Multi-objective optimization of functionally graded materials with temperature-dependent material properties. Mater. Des..

[13] Li K., Zhang M., Zhang Z., Jin P., Wang Y., Yan W., Zhu L., Zhang D.Z., Murr L.E. (2025). High performance realization of functionally graded materials based on integrated optimal design and additive manufacturing: A review. Int. Mater. Rev..

[14] El-Galy I.M., Saleh B.I., Ahmed M.H. (2019). Functionally graded materials classifications and development trends from industrial point of view. SN Appl. Sci..

[15] Ghazzawi S.M., Abdelrahman W.G. (2020). Static Analysis of Thick Functionally Graded Plates with Different Property Distribution Functions. Arab. J. Sci. Eng..

[16] Gautam M., Sharma P., Chaturvedi M. (2024). Modeling of FGM beam under an extended exponential law. Int. J. Interact. Des. Manuf. (IJIDeM).

[17] Balaraman P., Raj V.S.J., Sreehari V.M. (2022). Static and Dynamic Analysis of Re-Entry Vehicle Nose Structures Made of Different Functionally Graded Materials. Aerospace.

[18] Tian J., Jing G., Han X., Hu G., Huo S. (2021). Understanding the thermal problem of variable gradient functionally graded plate based on hybrid numerical method under linear heat source. Adv. Mech. Eng..

[19] Yevtushenko A., Topczewska K., Zamojski P. (2021). The Effect of Functionally Graded Materials on Temperature during Frictional Heating: Under Uniform Sliding. Materials.

[20] Yevtushenko A., Topczewska K., Zamojski P. (2023). Use of Functionally Graded Material to Decrease Maximum Temperature of a Coating–Substrate System. Materials.

[21] Sobamowo M.G., Oguntala G.A., Yinusa A.A. (2019). Nonlinear Transient Thermal Modeling and Analysis of a Convective-Radiative Fin with Functionally Graded Material in a Magnetic Environment. Model. Simul. Eng..

[22] Han D., Fan H., Yan C., Wang T., Yang Y., Ali S., Wang G. (2022). Heat Conduction and Cracking of Functionally Graded Materials Using an FDEM-Based Thermo-Mechanical Coupling Model. Appl. Sci..

[23] Nastasescu V. (2022). The Using of the Multilayer Plate Concept in the Calculus of Functionally Graded Plates. Appl. Sci..

[24] Noda N. (1991). Thermal stresses in materials with temperature-dependent properties. Appl. Mech. Rev..

[25] Dose G., Roccella S., Romanelli F. (2022). Engineering of a FGM Interlayer to Reduce the Thermal Stresses Inside the PFCs. Appl. Sci..

[26] Szlachetka O., Giorgio I. (2024). Heat conduction in multi-component step-wise FGMs. Contin. Mech. Thermodyn..

[27] Baytak T., Tosun M., Ipek C., Mollamahmutoglu C., Bulut O. (2024). Thermal Stress Analysis for Functionally Graded Plates with Modulus Gradation, Part II. Exp. Mech..

[28] Povstenko Y.Z. (2004). Fractional heat conduction equation and associated thermal stress. J. Therm. Stress..

[29] Jabbari M., Ghannad M., Nejad M.Z. (2016). Effect of Thickness Profile and FG Function on Rotating Disks Under Thermal and Mechanical Loading. J. Mech..

[30] Chen G., Zhai P.C., Zhang Q.J. (2003). Optimization of material composition of FGM coating under thermal loading by micro genetic algorithms. Mater. Sci. Forum.

[31] Damircheli M., Azadi M. (2011). Temperature and thickness effects on thermal and mechanical stresses of rotating FG-disks. J. Mech. Sci. Technol..

[32] Dbouk T. (2017). A review about the engineering design of optimal heat transfer systems using topology optimization. Appl. Therm. Eng..

[33] Subramaniam V., Dbouk T., Harion J.-L. (2018). Topology optimization of conductive heat transfer devices: An experimental investigation. Appl. Therm. Eng..

[34] Sha W., Xiao M., Wang Y., Huang M., Li Q., Gao L. (2024). Topology optimization methods for thermal metamaterials: A review. Int. J. Heat Mass Transf..

[35] Ikonen T.J., Marck G., Sóbester A. (2018). Topology optimization of conductive heat transfer problems using parametric L-systems. Struct. Multidisc. Optim..

[36] Meliga P., Abdel Nour W., Laboureur D., Serret D., Hachem E. (2024). Multi-Objective Topology Optimization of Conjugate Heat Transfer Using Level Sets and Anisotropic Mesh Adaptation. Fluids.

[37] Zhuang C., Xiong Z., Ding H. (2021). Temperature-constrained topology optimization of nonlinear heat conduction problems. J. Comput. Des. Eng..

[38] Giraldo-Londoño O., Mirabella L., Dalloro L., Paulino G.H. (2020). Multi-material thermomechanical topology optimization with applications to additive manufacturing: Design of main composite part and its support structure. Comput. Methods Appl. Mech. Eng..

[39] Pizzolato A., Sharma A., Maute K., Sciacovelli A., Verda V. (2019). Multi-scale topology optimization of multi-material structures with con-trollable geometric complexity—Applications to heat transfer problems. Comput. Methods Appl. Mech. Eng..

[40] Da D., Cui X., Long K., Cai Y., Li G. (2019). Multiscale concurrent topology optimization of structures and microscopic multi-phase materials for thermal conductivity. Eng. Comput..

[41] Guo Y., Wang Y., Wei D., Chen L. (2023). Multiscale concurrent topology optimization for thermoelastic structures under design-dependent varying temperature field. Struct. Multidiscip. Optim..

[42] Al Ali M., Shimoda M. (2025). Multiscale topology optimization for enhanced thermal management in lightweight laser-activated porous actuators. Optim. Eng..

[43] Deaton J.D., Grandhi R.V. (2014). A survey of structural and multidisciplinary continuum topology optimization: Post 2000. Struct. Multidiscip. Optim..

[44] Gersborg-Hansen A., Bendsøe M.P., Sigmund O. (2006). Topology optimization of heat conduction problems using the finite volume method. Struct. Multidiscip. Optim..

[45] Wu S., Zhang Y., Liu S. (2021). Transient thermal dissipation efficiency based method for topology optimization of transient heat conduction structures. Int. J. Heat Mass Transf..

[46] Das S., Sutradhar A. (2020). Multi-physics topology optimization of functionally graded controllable porous structures: Application to heat dissipating problems. Mater. Des..

[47] Qureshi Z.A., Al-Omari S.A.B., Elnajjar E., Al-Ketan O., Al-Rub R.A. (2022). On the effect of porosity and functional grading of 3D printable triply periodic minimal surface (TPMS) based architected lattices embedded with a phase change material. Int. J. Heat Mass Transf..

[48] Senthil S., Pelletier J.L. (2007). Multi-objective optimization of functionally graded thick shells for thermal loading. Compos. Struct..

[49] Correia V.M.F., Madeira J.A., Araújo A.L., Soares C.M.M. (2019). Multiobjective optimization of functionally graded material plates with thermo-mechanical loading. Compos. Struct..

[50] Nayak P., Armani A. (2022). Optimal Design of Functionally Graded Parts. Metals.

[51] Li Y., Feng Z., Hao L., Huang L., Xin C., Wang Y., Bilotti E., Essa K., Zhang H., Li Z. (2020). A Review on Functionally Graded Materials and Structures via Additive Manufacturing: From Multi-Scale Design to Versatile Functional Properties. Adv. Mater. Technol..

[52] Shir O.M., Bäck T. (2006). Niche radius adaptation in the CMA-ES niching algorithm. Parallel Problem Solving from Nature-PPSN IX.

[53] Ulrich T., Bader J., Thiele L. (2010). Defining and optimizing indicator-based diversity measures in multiobjective search. Parallel Problem Solving from Nature-PPSN IX.

[54] Pal M., Bandyopadhyay S. (2021). Decomposition in decision and objective space for multi-modal multi-objective optimization. Swarm Evol. Comput..

[55] Segura C., Castillo J.C., Schütze O. (2023). The Importance of Diversity in the Variable Space in the Design of Multi-Objective Evolutionary Algorithms. Appl. Soft Comput..

[56] Liu Q., Zou J., Yang S., Zheng J. (2022). A multiobjective evolutionary algorithm based on decision variable classification for many-objective optimization. Swarm Evol. Comput..

[57] Miyamoto Y., Kaysser W.A., Rabin B.H., Kawasaki A., Ford R.G. (1999). Functionally Graded Materials: Design, Processing and Applications.

[58] Zienkiewicz O.C., Taylor R.L., Zhu J.Z. (2013). The Finite Element Method: Its Basis and Fundamentals.

[59] Martínez-Pañeda E. (2019). On the Finite Element Implementation of Functionally Graded Materials. Materials.

[60] Vel S.S., Batra R.C. (2002). Exact solution for thermoelastic deformations of functionally graded thick rectangular plates. AIAA J..

[61] Kim J.-H., Paulino G.H. (2002). Isoparametric Graded Finite Elements for Nonhomogeneous Isotropic and Orthotropic Materials. J. Appl. Mech..

[62] Nguyen-Xuan H., Tran L.V., Thai C.H., Kulasegaram S., Bordas S.P.A. (2014). Isogeometric analysis of functionally graded plates using a refined plate theory. Compos. Part B Eng..

[63] Aubertin P., Réthoré J., de Borst R. (2010). A coupled molecular dynamics and extended finite element method for dynamic crack propagation. Int. J. Numer. Methods Eng..

[64] Reddy J.N. (2004). Mechanics of Laminated Composite Plates and Shells: Theory and Analysis.

[65] Jha D.K., Kant T., Singh R.K. (2013). A critical review of recent research on functionally graded plates. Compos. Struct..

[66] Barth T., Herbin R., Ohlberger M. (2018). Finite Volume Methods: Foundation and Analysis. Encyclopedia of Computational Me-chanics.

[67] Versteeg H.K., Malalasekera W. (2007). An Introduction to Computational Fluid Dynamics: The Finite Volume Method.

[68] Charoensuk J., Vessakosol P. (2010). A high order control volume finite element procedure for transient heat conduction analysis of functionally graded materials. Heat Mass Transf..

[69] Gong J., Xuan L., Ming P., Zhang W. (2013). An Unstructured Finite-Volume Method for Transient Heat Conduction Analysis of Multilayer Functionally Graded Materials with Mixed Grids. Numer. Heat Transf. Part B Fundam..

[70] Ghia U., Ghia K., Shin C. (1982). High-Re solutions for incompressible flow using the Navier-Stokes equations and a multigrid method. J. Comput. Phys..

[71] Cavalcante M.A., Marques S.P., Pindera M.-J. (2008). Computational aspects of the parametric finite-volume theory for functionally graded materials. Comput. Mater. Sci..

[72] Garg S., Pant M. (2018). Meshfree methods: A comprehensive review of applications. Int. J. Comput. Methods.

[73] Silling S., Lehoucq R. (2010). Peridynamic Theory of Solid Mechanics. Adv. Appl. Mech..

[74] Bautista V., Shahbazian B., Mirsayar M. (2025). Mixed-Mode Timoshenko-Based Peridynamics for Dynamic Crack Propagation in Functionally Graded Materials. Fatigue Fract. Eng. Mater. Struct..

[75] Nikolaev P., Jivkov A.P., Margetts L., Sedighi M. (2024). Non-Local Formulation of Heat Transfer with Phase Change in Domains with Spherical and Axial Symmetries. J. Peridyn. Nonlocal Model..

[76] Gu X., Zhang Q., Madenci E. (2019). Refined bond-based peridynamics for thermal diffusion. Eng. Comput..

[77] Hughes T., Cottrell J., Bazilevs Y. (2005). Isogeometric analysis: CAD, finite elements, NURBS, exact geometry and mesh refinement. Comput. Methods Appl. Mech. Eng..

[78] Cottrell J.A., Hughes T.J.R., Bazilevs Y. (2009). Isogeometric Analysis: Toward Integration of CAD and FEA.

[79] Mykhaskiv O., Banović M., Auriemma S., Mohanamuraly P., Walther A., Legrand H., Müller J.-D. (2018). NURBS-based and parametric-based shape optimization with differentiated CAD kernel. Comput. Des. Appl..

[80] Duh U., Shankar V., Kosec G. (2024). Discretization of Non-uniform Rational B-Spline (NURBS) Models for Meshless Isogeometric Analysis. J. Sci. Comput..

[81] Belytschko T., Lu Y.Y., Gu L. (1994). Element-free Galerkin methods. Int. J. Numer. Methods Eng..

[82] Liu G.R., Gu Y.T. (2005). An Introduction to Meshfree Methods and Their Programming.

[83] Atluri S.N., Zhu T. (1998). A new Meshless Local Petrov-Galerkin (MLPG) approach in computational mechanics. Comput. Mech..

[84] Wang H., Qin Q.-H., Kang Y.-L. (2006). A meshless model for transient heat conduction in functionally graded materials. Comput. Mech..

[85] LeFloch P.G., Mercier J. (2020). The Transport-based Mesh-free Method: A Short Review. Wilmott.

[86] Suresh S., Mortensen A. (1998). Fundamentals of Functionally Graded Materials: Processing and Thermomechanical Behaviour of Graded Metals and Metal-Ceramic Composites.

[87] Reddy J.N., Chin C.D. (1998). Thermomechanical Analysis of Functionally Graded Cylinders and Plates. J. Therm. Stress..

[88] Reddy J.N. (2000). Analysis of functionally graded plates. Int. J. Numer. Methods Eng..

[89] Birman V., Byrd L.W. (2007). Modeling and Analysis of Functionally Graded Materials and Structures. Appl. Mech. Rev..

[90] Liew K., Kitipornchai S., Zhang X., Lim C. (2003). Analysis of the thermal stress behaviour of functionally graded hollow circular cylinders. Int. J. Solids Struct..

[91] Hoe A., Barako M.T., Tamraparni A., Zhang C., Elwany A., Felts J.R., Shamberger P.J. (2022). Objective oriented phase change material composite heat sink design. Appl. Therm. Eng..

[92] Arshad A., Jabbal M., Faraji H., Talebizadehsardari P., Bashir M.A., Yan Y. (2022). Thermal performance of a phase change material-based heat sink in presence of nanoparticles and metal-foam to enhance cooling performance of electronics. J. Energy Storage.

[93] Ye F., Dong Y., Opolot M., Zhao L., Zhao C. (2024). Assessment of Thermal Management Using a Phase-Change Material Heat Sink under Cyclic Thermal Loads. Energies.

[94] Zou K., Bai P., Li K., Luo F., Liang J., Lin L., Zhang G. (2024). Electronic cooling and energy harvesting using ferroelectric polymer composites. Nat. Commut..

[95] Chen Y., Zhang M., Su Y., Zhou Z. (2021). Coupling Analysis of Flexoelectric Effect on Functionally Graded Piezoelectric Cantilever Nanobeams. Micromachines.

[96] Li X., Wilson C.T., Zhang L., Bhatia B., Zhao L., Leroy A., Brandt O., Orta-Guerra R., Youngblood J.P., Trice R.W. (2022). Design and modeling of a multiscale porous ceramic heat exchanger for high temperature applications with ultrahigh power density. Int. J. Heat Mass Transf..

[97] Lee W.Y., Stinton D.P., Berndt C.C., Erdogan F., Lee Y., Mutasim Z. (1996). Concept of Functionally Graded Materials for Advanced Thermal Barrier Coating Applications. J. Am. Ceram. Soc..

[98] Boggarapu V., Gujjala R., Ojha S., Acharya S., Chowdary S., kumar Gara D. (2021). State of the art in functionally graded materials. Compos. Struct..

[99] Şafak İ., Şenyiğit E., Güneş S., Doğmaz M.A. (2025). Investigation of heat transfer in functionally graded annular fins under natural convection using Taguchi methods. J. Therm. Anal. Calorim..

[100] Wang J.P., Chen G., Zhai P.C. (2005). Optimization of Material Composition of FGM Coating under Steady Heat Flux Loading by Micro-Genetic Algorithms. Mater. Sci. Forum.

[101] Tong L., Liu J., Yi B., Liu L. (2024). Topology Optimization of Functionally Graded Structure for Thermal Management of Cooling Plate. Appl. Sci..

[102] Chen F.L., Lin Q.L., Yin H.M. Thermo-mechanical behavior of a novel functionally graded material panel. Proceedings of the 15th Biennial ASCE Conference on Engineering, Science, Construction, and Operations in Challenging Environments.

